# RNA splicing: a split consensus reveals two major 5′ splice site classes

**DOI:** 10.1098/rsob.240293

**Published:** 2025-01-15

**Authors:** Matthew T. Parker, Sebastian M. Fica, Gordon G. Simpson

**Affiliations:** ^1^ Max Planck Institute for Plant Breeding Research, Cologne, Germany; ^2^ Department of Biochemistry, University of Oxford, Oxford, UK; ^3^ School of Life Sciences, University of Dundee, Dundee, UK

**Keywords:** splicing, METTL16, SNRNP27K, m^6^A, T-loop, ReNU syndrome

## Abstract

The established consensus sequence for human 5′ splice sites masks the presence of two major splice site classes defined by preferential base-pairing potentials with either U5 snRNA loop 1 or the U6 snRNA ACAGA box. The two 5′ splice site classes are separable in genome sequences, sensitized by specific genotypes and associated with splicing complexity. The two classes reflect the commitment to 5′ splice site usage occurring primarily during 5′ splice site transfer to U6 snRNA. Separating the human 5′ splice site consensus into its two major constituents can help us understand fundamental features of eukaryote genome architecture and splicing mechanisms and inform treatment design for diseases caused by genetic variation affecting splicing.

**Figure:**
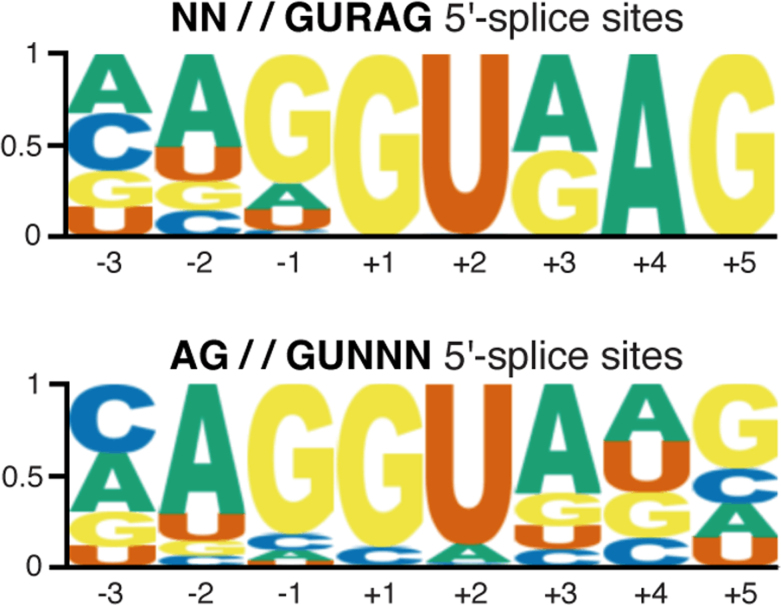

## 1. Introduction

A defining feature of eukaryotes is that their genes can be split into different pieces, which are spliced together in messenger RNA [1,2]. Alternative splicing generates multiple transcripts from a single gene that can diversify the proteins made, determine the regulatory fate of mRNA and influence phenotypic traits [3]. Consistent with this, patterns of alternative splicing complexity differ between species and can facilitate adaptation and contribute to species divergence [4]. Although most protein-coding genes in humans are alternatively spliced, in the budding yeast *Saccharomyces cerevisiae*, most genes lack introns, and alternative splice site selection is rare. While evolutionary conservation and contrasts illuminate splicing mechanisms [2], the processes that drive change in splicing complexity are unclear. Mutations that disrupt splicing cause specific human diseases [5]. Recent clinical application of treatments for some of these conditions demonstrates that understanding splicing can lead to novel therapeutic approaches [6].

## 2. The discovery of pre-mRNA splicing and splice site consensus sequences

Split genes were first reported in 1977 after analysis of adenovirus 2 mRNAs expressed in human cells [7,8]. In early 1978, commenting on these and subsequent discoveries of eukaryote genes expressed in pieces, Walter Gilbert suggested naming the regions lost from mature messenger RNA as ‘introns’ and the regions that will be expressed as ‘exons’ [9]. Later that year, Pierre Chambon and colleagues described the first alignment of different intron sequences [10]. They established the principle of unique excision-ligation points common to all intron–exon boundaries, suggesting that the 5′ end of introns was always defined with GU, whereas the 3′ ends were always defined with AG. As more intron sequences were uncovered, further common features were detected and a consensus was defined. The consensus sequence comprises the nucleotide most commonly found at each position. For example, the 5′ splice site consensus was described in a succession of papers around this time as AG//GUAAGU (// denotes the cleavage site) [11], AG//GUAAGUA [12], (A/C)AG//GUAAGU [13] and AG//GURAG [14]. In 1982, Steve Mount, then a graduate student in Joan Steitz’s lab, published the first paper dedicated exclusively to cataloguing splice junction sequences and defined a 5′ splice site consensus (A/C)AG//GURAGU based on the analysis of 139 introns [15]. The subsequent availability of the human genome sequence and global RNA-sequencing analyses did not fundamentally change this consensus.

## 3. Spliceosomes splice pre-mRNA

The identification of consensus sequences defining intron boundaries suggested that there must be a common mechanism for their recognition. Joan Steitz and colleagues first proposed and demonstrated that the uridylate-rich small nuclear RNA (UsnRNA) U1 base paired with the 5′ splice site and that other UsnRNAs probably functioned in splicing [13,16]. Genetic suppression of compensatory base-pair mutations was later used to show that base-pairing of the 5′ end of U1 snRNA to the 5′ splice site was necessary but not sufficient for the splicing of the adenovirus *E1A* gene [17]. Later, related approaches demonstrated that loop 1 of U5 snRNA base paired to exon sequence upstream of the 5′ splice site and that this was important for 5′ splice site selection [18,19]. Furthermore, the ACAGA box of U6 snRNA base paired to intron sequence downstream of the cleavage site and this was also important for 5′ splice site selection [20–22]. Subsequent progress over the ensuing years has led to a detailed molecular mechanism for splicing, culminating with the direct visualization of splicing snapshots through electron cryo-microscopy (cryo-EM) [23]. Much of this basic mechanism came from the biochemical characterization of splicing in humans and genetic and biochemical approaches using *S. cerevisiae*.

**Figure 1.**
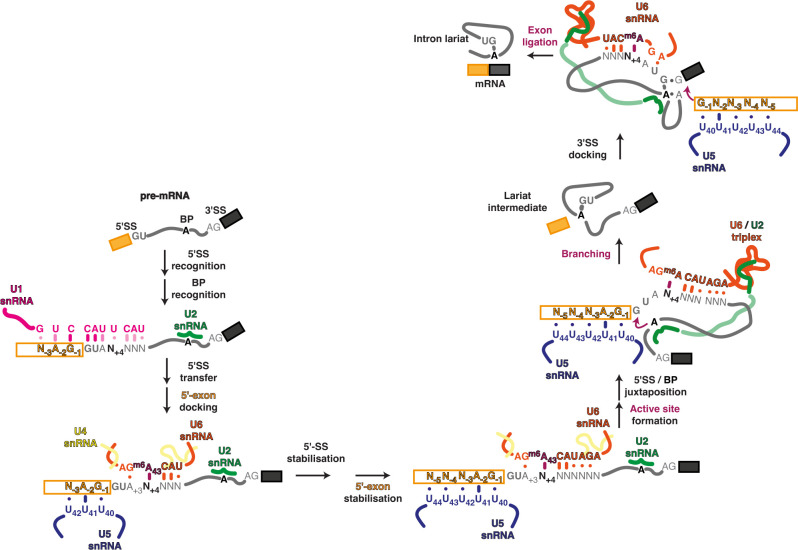
Splicing pathway. Initial recognition of the 5′-exon and 5′ splice site through extensive pairing to the U1 snRNA is followed by a likely stepwise hand-off to the U5 and U6 snRNAs, respectively. Following U1 dissociation, the newly formed U5 and U6 pairings are stabilized and extended. Subsequent dissociation of the U4 snRNA allows formation of the active site triplex and docking of the branch point adenosine (BP-A) in the active site for branching. Remodelling of the resulting lariat-intermediate is required for docking of the 3′ splice site in the active site, where it can be attacked by the cleaved 5′-exon to form the mRNA. Variable, non-Watson–Crick pairings are denoted by a dot. Note that pairing to U6 snRNA m^6^A43 caps the end of the newly formed 5′ splice site helix throughout the catalytic stage and can thus influence both branching and exon ligation.

Splicing is mediated by spliceosomes—dynamic assemblies of five UsnRNAs and more than 100 proteins. Specific spliceosome complexes that mediate different splicing stages are defined by remodelled interactions between the snRNAs and the pre-mRNA (figure 1). In humans, U1 snRNA can identify potential 5′ splice sites in pre-mRNA by base pairing interactions that span the exon sequence immediately upstream and the intron sequence immediately downstream of the 5′ splice site [24]. The branch site and 3′ splice site are typically recognized by cooperative interactions of U2 snRNA, which base-pairs to sequences around the intron branch site with auxiliary SF1 and U2AF proteins [25]. A preassembled U4/U6.U5 tri-snRNP subsequently joins the spliceosome. The 5′ splice sites are then transferred to the U6 snRNA ACAGA box, which binds to intron sequences immediately downstream of the 5′ splice site, and to U5 snRNA loop 1, which binds exon sequences immediately upstream. Cryo-EM analyses reveal that in humans, but not *S. cerevisiae*, 5′ splice site transfer is uncoupled from active site formation [25,26]. In humans, the formation and stabilization of the U6/5′ splice site helix seems to be a prerequisite for subsequent ATP-dependent spliceosome rearrangements that drive active site formation [27]. The active site, with two metal ions, is formed by molecular interactions between U2 and U6 snRNA [28] after U6 is freed from its U4 snRNA chaperone. When the branch site adenosine docks in the active site, the branching reaction occurs, resulting in cleavage of the 5′ splice site and the formation of a branched intron-lariat intermediate. The 5′ exon remains in the active site, but the intron lariat is removed and replaced by the 3′ splice site for the exon-ligation reaction (figure 1). The ligated exons are then released as spliced mRNA. U6 snRNA catalyses both splicing reactions by positioning the divalent metal ions that stabilize each reaction [28]. Therefore, the spliceosome shares common catalytic mechanisms, and probably evolutionary origins, with self-splicing group II introns [2,28,29].

A major difference between human and *S. cerevisiae* spliceosomes results from how introns are organized and defined. Not only do most *S. cerevisiae* genes lack introns, but they also rarely encode more than one intron per gene. In contrast, humans have many introns per gene, and introns can be many kilobases in length. These distinctions probably explain why in humans, but not *S. cerevisiae*, some introns are recognized for splicing following the definition of flanking exon sequences [30]. Splicing following exon definition requires a cross-exon to cross-intron switch during spliceosome assembly [27,31].

## 4. Two 5′ splice site classes revealed by genome sequence analyses

Consensus aligning of all intron sequences can overlook differing 5′ splice site sequence preferences within subpopulations. Despite this, early computational genome analyses hinted at different 5′ splice site sequence features. U1 snRNA was considered the defining selector of 5′ splice sites, but other analyses and more recent studies emphasize the roles of U5 and U6 snRNAs in 5′ splice site selection. As a result, a clearer mechanistic explanation of the different features of 5′ splice site consensus sequences is possible.

In the late 1990s, interpretation of the then-emerging human genome sequence required new computational tools to predict genes and introns. GENSCAN software, developed at that time, revealed common features of 5′ splice sites, indicating that they could be sub-classified [32]. A 5′/3′ compensation effect was identified, in which matches to consensus nucleotides at nearby positions on the same side of the intron/exon junction were positively associated, while poor matching on one side of the junction was almost always compensated by stronger matching on the other. For example, a particularly strong association was detected between the exonic −1 and intronic +5 positions. These 5′ splice site sequence features were interpreted in terms of U1 snRNA interaction potential. Without conclusive evidence; however, they could be explained by interaction preferences at other stages of the splicing reaction. Subsequent studies, made as more genome sequences became available, reinforced the idea of different 5′ splice site features, retaining the interpretation based on U1 snRNA interactions and renaming this phenomenon seesaw linkage [33,34].

**Figure 2.**
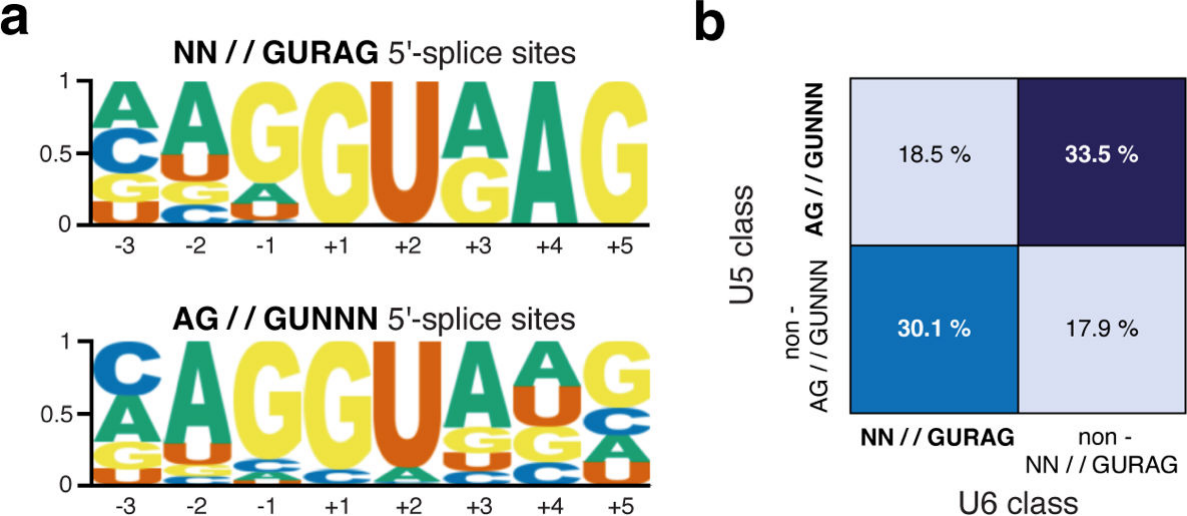
Sequence and relative usage of the two classes of 5′ splice site. (*a*) Logograms for experimentally annotated 5′ splice site classes used in humans. (*b*) Contingency tables for classes of 5′ splice sites used in humans.

A more recent human genome analysis revealed that only 53.6% of 5′ splice sites have an NN//GURAG consensus sequence, while 45.1% do not and have an AG//GUNNN consensus sequence instead [35]. Analysis of the annotated 5′ splice sites of *Arabidopsis thaliana, Caenorhabditis elegans*, *Drosophila melanogaster* and *Danio rerio* genomes revealed a similar pattern of separable sub-classes of 5′ splice sites [36]. These analyses suggest that two classes of 5′ splice sites exist: NN//GURAG and AG//GUNNN, which occur in metazoans in an approximately 1 : 1 ratio (figure 2) [36]. Since the sequence composition of 5′ splice sites retrieved in this way is not mutually exclusive, this is not a precisely defined ratio (figure 2). However, these sub-classes of 5′ splice sites tend to be anti-correlated and rarely co-occur [36].

The two 5′ splice site sub-classes, NN//GURAG and AG//GUNNN, would still allow significant pairing to U1 snRNA, and thus, their differential pattern of occurrence probably reflects interactions with other elements. Indeed, the two classes can be defined by a preferential base-pairing interaction potential with either U5 snRNA loop 1 (AG//GUNNN) or the U6 snRNA ACAGA box (NN//GURAG) [36], which can simultaneously explain 5′/3′ compensation and seesaw linkage. For example, a G consensus at −1 allowing any nucleotide at position +5 [34] corresponds to the AG//GUNNN sub-class, with a strong interaction potential with U5 snRNA loop 1. Likewise, the +5G consensus allowing any nucleotide at −1 [34] can be explained by the NN//GURAG sub-class, with a strong interaction potential with the U6 snRNA ACAGA box. Consistent with this interpretation, other computational analyses have suggested that the seesaw linkage of the −3 exonic position with intronic sequences is better explained by interactions with U5 snRNA than U1 snRNA [37]. In summary, two major classes of 5′ splice sites can be separated in the genome sequences of diverse species [36].

## 5. Two 5′ splice site classes revealed by genetic disruption of spliceosomes

The two major sub-classes of U2-dependent introns’ 5′ splice sites were revealed orthogonally by analysing genotypes of different species following disruption of specific spliceosome components. Sequencing of mRNAs produced by these disrupted genotypes revealed a recurring phenotype: a switch away from the selection of 5′ splice sites with a strong interaction potential with the U6 snRNA ACAGA box to sites with a stronger interaction potential with U5 snRNA loop 1 in the upstream exon, coupled with a major change in preference for the base at the +4 position. Therefore, specific genetic backgrounds sensitize these two splice site classes.

The sensitization of two splice site classes was first reported for genotypes defective in U6 snRNA modification [36,38,39]. The central adenosine of the human U6 snRNA ACAGA box (U6 m^6^A43) is methylated at the N6 position by METTL16 [40,41]. Global RNA-sequencing analysis of *Arabidopsis* METTL16 (*fiona1*) mutants revealed thousands of splicing changes that could be explained by altered patterns of 5′ splice site selection: 5′ splice sites used less in the *fio1* mutant had a stronger interaction potential with the U6 snRNA ACAGA box and tended to have an A at the +4 position, but alternative sites used more in the mutant tended to have +4U and/or a stronger consensus sequence in the upstream exon [36]. Related splicing changes were found in *C. elegans* METTL16 orthologue (*mett10*) mutants and human METTL16 knockdown cell lines [39]. Deletion of the METTL16 orthologue from *Schizosaccharomyces pombe* resulted in less efficient splicing of introns that tend to have a +4A at the 5′ splice site, while introns that were spliced more efficiently were enriched for +4U [38]. In *Arabidopsis fio1*, *S. pombe mtl16Δ* and *C. elegans mett10* mutants, and human METTL16 knockdown cell lines, increased alternative 5′ splice site usage also occurs at sites with stronger interaction potential with U5 snRNA loop 1 in the upstream exon [36,38,39]. The expression of compensatory U5 snRNA loop 1 mutants in *S. pombe mtl16Δ*, which optimizes base-pairing interactions with upstream exon sequences, rescues inefficient splicing at targeted sites compromised in *mtl16Δ* mutants [38]. Therefore, splice sites sensitive to disruption of U6 snRNA ACAGA box interactions can be rescued by compensatory interactions with U5 snRNA loop 1.

Splicing changes related to those in METTL16 orthologue mutants are seen in *C. elegans* mutants defective in an orthologue of human SNRNP27K (SNRP-27). SNRNP27K is a largely disordered spliceosomal protein that comprises an N-terminal SR domain and a conserved C-terminal domain [42,43]. A *C. elegans* mutant isolated in a screen for factors that modulate splicing fidelity has a methionine to threonine change at position 141 of the SNRP-27 C-terminal domain [43]. RNA-sequencing analysis of *snrp-27* M141T mutants revealed a switch of splicing away from 5′ splice sites with +4A [43]. A reanalysis of these data, which revealed many more splicing changes, shows significant overlap with sites sensitive to loss of the *C. elegans* METTL16 orthologue, mett10 [39]. Like METTL16 orthologue mutants in different species, splice site switches in *snrp-27* M141T mutants involve increased usage of sites with stronger interaction potential with U5 snRNA loop 1 in the upstream exon [39].

Two 5′ splice site classes are also sensitized by variants in the human *RNAU4-2* gene encoding U4 snRNA that cause a frequent neurodevelopmental disorder called ReNU syndrome [44–46]. The U6 snRNA ACAGA box is projected as a mobile loop towards U1 snRNP in pre-B complexes, partly by a T-loop structure (previously termed quasi-pseudo knot) formed between U4 and U6 snRNA [26]. RNA-sequencing data from individuals with *RNAU4-2* variants that disrupt the T-loop reveal a switch in 5′ splice site usage away from sites with a strong interaction potential with the U6 snRNA ACAGA box (+3, +4 and +5 positions of the 5′ splice site) to sites with a weaker U6 snRNA interaction potential and a stronger interaction with U5 snRNA loop 1 in the upstream exon [45,46].

Finally, the analysis of human LUC7 proteins’ role in splicing reveals a highly similar sensitization of two 5′ splice site classes [47]. Humans (and many other species) have three LUC7-related genes; *LUC7L*, *LUC7L2* and *LUC7L3*. In contrast, *S. cerevisiae* has a single *LUC7* gene that encodes a splicing factor, which stabilizes the U1/5′ splice site helix in pre-B spliceosomal complexes [48]. A reanalysis of combined RNA-sequencing data sets of human LUC7L2 knockout and LUC7L, LUC7L2 and LUC7L3 knockdown cell lines revealed differential sensitivity of specific 5′ splice sites to individual LUC7 paralogues [47]. Disrupted LUC7L and LUC7L2 function results in a switch away from the selection of 5′ splice sites with a strong U6 snRNA ACAGA box interaction potential consistent with a potential role in stabilizing the U1/5′ splice site interaction, as in *S. cerevisiae*. In contrast, the knockdown of LUC7L3 results in a global switch away from selecting 5′ splice sites with a strong interaction potential with U5 snRNA loop 1.

In summary, the impact of genetic disruption of U6 snRNA m^6^A modification, SNRNP27K orthologue and LUC7 paralogues in different species and U4/U6 T-loop sequence variation in humans is related because each of these perturbations sensitizes two splice site classes. These data, coupled with the two major sub-classes of 5′ splice site sequence identified by genome analyses, underline the idea that there are two classes of 5′ splice site recognized by preferential interactions with either U5 snRNA loop 1 or the U6 snRNA ACAGA box [36].

## 6. The two classes of 5′ splice site impact co-ordinated transfer of the 5′ splice site

**Figure 3.**
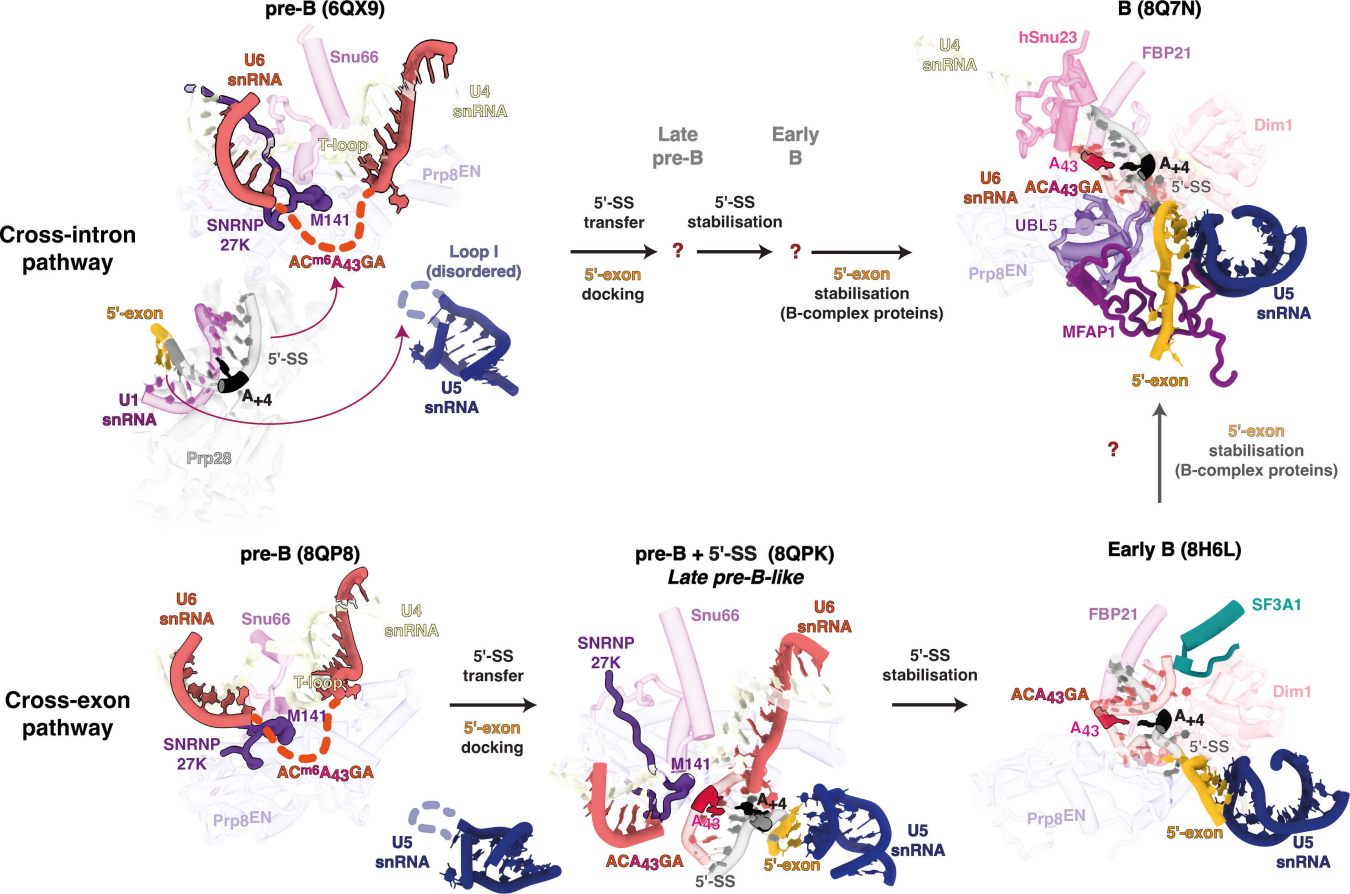
Structures of key intermediates during 5′ splice site transfer to the U5 and U6 snRNAs. The upper part of the illustration depicts intermediates obtained following assembly across an *AdML*-derived intron. Only the starting pre-B and final B states were observed experimentally. The additional intermediates are suggested, as inferred from complexes observed in the cross-exon pathway. Note that in pre-B, the ACAGAGA region of U6 and loop I of U5 snRNA are disordered in the cryo-EM structures. The lower part shows intermediates observed following assembly on an exon flanked by an upstream 3′ splice site and a downstream 5′ splice site, in the so-called cross-exon pathway. The key middle intermediate after 5′ splice site transfer was obtained by chasing a purified pre-B dimer complex with an oligo mimicking the 5′ splice site in *trans* [27] but mimics, and is essentially superimposable onto, a similar so-called ‘late pre-B’ state obtained by classification of intermediates from a cross-exon assembly reaction in extract [31]. The shown early B state was obtained from the same cross-exon reaction in extract. A B-like complex essentially identical to the B complex observed in the cross-intron pathway, but lacking ordered density for MFAP1 and UBL5 was also observed by chasing the purified pre-B cross-exon dimer in extract in the presence of ATP and a 5′ splice site in *trans*, suggesting that the two pathways converge at the B complex stage, as indicated in the figure. Key RNA and protein residues are modelled in atomic detail, and the PDB model codes are indicated for each state. Dim1 is present throughout, both before and after transfer, but is omitted in the pre-B states for clarity.

It is striking that, so far, the spliceosomal components found to sensitize two 5′ splice site classes when mutated or downregulated all appear to function during the transfer of candidate 5′ splice sites from U1 snRNA to U5 and U6 snRNA prior to spliceosome activation [36,39,45–47,49]. These events have been illuminated in new detail by recent cryo-EM structures of the cross-exon to cross-intron switch, which reveal intermediate stages in 5′ splice site transfer [27,31,50]. The emerging functional and structural data indicate that transfer of the 5′ splice site from U1 to U5 and U6 snRNAs is a coordinated stepwise process allowing for regulation before commitment to a selected 5′ splice site. Initial docking, dependent on weaker RNA–RNA interactions, is progressively stabilized by protein factors that abut the newly formed RNA helices in both the pre-B and B complexes (figure 3). The final sensing of a strongly docked 5′ splice site is then relayed to Brr2 to initiate spliceosome activation. The concept of stepwise commitment to 5′ splice site usage occurring primarily during hand-off of the 5′ splice site from U1 to U6 snRNA is supported by PRP28-mediated proofreading of 5′ splice site usage [51]. PRP28 promotes the transfer of 5′ splice sites from U1 to U6 snRNA and appears to sense the stability of the U6/5′ splice site helix. This proofreading mechanism would also explain the need for additional stabilization of the U5 and U6 snRNA interactions by protein factors associated with B complex.

The new structural data reveal how closely oriented in time and space the T-loop, m^6^A-modified U6 snRNA ACAGA box and SNRNP27K M141 are during 5′ splice site transfer to U5 and U6 snRNA [27,31,50]. The T-loop functions to project the U6 snRNA ACAGA box flexibly into space for 5′ splice site docking. Insertion variants in human *RNU4-2* [44–46] may alter U4/U6 T-loop formation and/or stabilization, affecting U6 snRNA ACAGA box positioning and hence splice site usage. The importance of ACAGA positioning by the T-loop is underscored by the fact that RBM42, which stabilizes the U4/U6 T-loop in the pre-B complex, is displaced following T-loop unwinding after the U6/5′ splice site helix has formed in the B complex [26].

**Figure 4.**
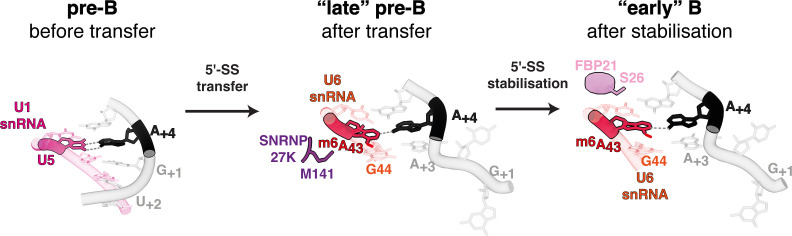
RNA interactions involving 5′ splice site A+4 during initial recognition and transfer. Note the switch from strong Watson–Crick pairing to U1 snRNA to weaker non-canonical pairing with U6 m^6^A43. This feature probably creates an equilibrium between the pre-B and B complexes, which allows for increased plasticity of 5′ splice site selection.

**Figure 5.**
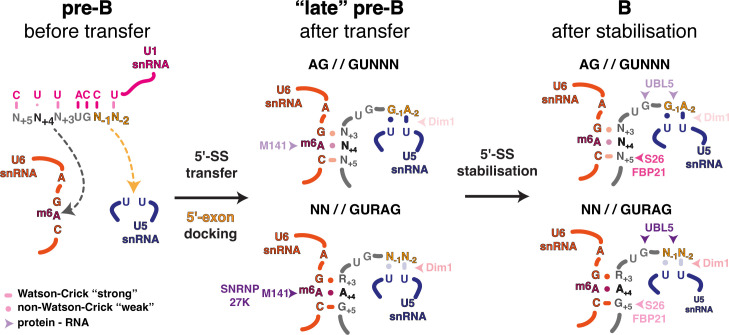
Key RNA interactions during 5′ splice site transfer. The two main classes of 5′ splice site engage in stronger interactions with either the U6 or U5 snRNAs. Specific protein side chains alternatively stabilize pairing to each snRNA, depending on the strength of interactions with each 5′ splice site class. The proposed importance of such stabilizing interactions is indicated by the relative opacity of the protein label.

The recurrent connection of METTL16 orthologue mutants to the 5′ splice site +4 position is explained by cryo-EM structures of human B complex spliceosomes, which show that the central adenosine of the U6 snRNA ACAGA box faces the +4 position in a trans-Hoogsteen sugar edge interaction [36,52] (figures 3–5). Biophysical studies indicate that in short helices m^6^A : A pairs are stronger than A : A, and that m^6^A : U pairs are destabilizing because the N6 methylamino group orientates into the major groove, pushing a short helix apart [53,54]. Hence, in the absence of U6 snRNA m^6^A modification, Watson–Crick A : U pairs are favoured at the +4 position, reflecting this alternative splice site usage feature in METTL16 orthologue mutants [36,38,39]. Biophysical studies also reveal that m^6^A strongly stabilizes base stacking at helix ends [54]. U6 m^6^A43 may therefore help to cap or stabilize the end of the newly formed U6/5′ splice site helix during the early stages of 5′ splice site transfer (figures 3 and 4) [36]. The 5′ end of the intron probably initiates transfer to form the U6/5′ splice site helix, yet the +3 base of the 5′ splice site—usually an A or a G—can only interact weakly with the corresponding G44 of U6 snRNA (figure 4) and is thus not fully aligned to stack on A+4. Degenerate 5′ splice site sequences present downstream of A+4 in many introns make the initial helix formed with the U6 snRNA ACAGA box relatively weak, short and mainly comprised of non-canonical RNA–RNA interactions requiring stabilization. The stability of the U6/5′ splice site helix is subsequently enhanced as it grows through additional non-canonical RNA–RNA interactions and by SNU23 binding [27]. The new structural data also explain the connection of SNRNP27K M141 to the +4A position [39,49]. SNRNP27K is present when the U6/5′ splice site helix is first formed (figure 3, lower part) with M141 probably stabilizing U6 m^6^A43 pairing at the +4 position [27,31] (figure 4), potentially through some π stacking with A43 (however, the M141 side chain cannot be modelled accurately in the available maps). SNRNP27K is then displaced prior to spliceosome activation and unwinding of U4/U6 by Brr2 (figure 3, upper-right part) [27]. *C. elegans snrp-27* mutants do not wholly phenocopy *mett10* mutants because they also show a switch away from sites with +3G to +3A, which may reflect the role of the +3 position in stabilizing initial pairing at the end of the U6/5′ splice site helix in the absence of SNRNP27K.

The exact function of the human LUC7 proteins is not clear. Analysis of human LUC7L, L2 and L3 protein interactions suggests that they are associated with components of U1 snRNP and A and B spliceosome complexes [55,56] and may be present during the transfer of the 5′ splice site from U1 to U6 snRNA [56]. Nonetheless, unlike in *S. cerevisiae*, human LUC7 proteins are not directly observed to bind the U1/5′ splice site helix in purified pre-B spliceosomes. Instead, recent analysis of the cross-exon to cross-intron conversion suggests LUC7 proteins may tether SR proteins and the U1 snRNP, potentially acting indirectly to affect initial 5′ splice site recognition [27].

It is also striking how different 5′ splice site transfer is in humans compared with *S. cerevisiae*. In *S. cerevisiae*, relatively invariant 5′ splice sites are recognized by U1 snRNA pairing and the transfer to U6 snRNA is directly coupled to active site formation [23,26]. In contrast, the uncoupling of human 5′ splice site transfer to U6 snRNA from active site formation provides opportunities for the spliceosome to determine 5′ splice site usage. Indeed, it has been proposed that this fundamental distinction between human and *S. cerevisiae* spliceosomes may provide a mechanism, which allows alternative 5′ splice site usage in humans [25]. Transfer distinctions between human and *S. cerevisiae* spliceosomes are clear. For example, RBM42, SNRNP27K, the U4/U6 snRNA T-loop and m^6^A modification of the U6 snRNA ACAGA box have all been lost from *S. cerevisiae* [26,49].

An important question on 5′ splice site transfer and the two splice site classes relates to the role of U1 snRNA. Cryo-EM snapshots of human spliceosomes capture features of the transfer of 5′ splice sites from U1 to U5 and U6 snRNA [26]. However, human U1 snRNA is not required for splicing in certain contexts: for example, splicing in human *in vitro* splicing extracts can proceed without U1 snRNA if more SR proteins are added [57–59]; *in vitro* splicing of synthetic RNAs, in which a short 5′ splice site RNA oligonucleotide is added in *trans*, does not require U1 snRNA binding to the 5′ splice site [60]; U6 snRNA can select different 5′ splice sites to those identified by U1 snRNA binding *in vivo* [22,24,61,62]; U4/U6.U5 tri-snRNPs can be recruited to complexes formed at exon splicing enhancers, with 5′ splice site selection being dependent on U6 snRNA ACAGA box interactions rather than U1 snRNA [63]. Splicing regulation may thus occur primarily at the point of U4/U6.U5 tri-snRNP recruitment and hence the selection of 5′ splice sites by U6 snRNA ACAGA box pairing rather than earlier during initial U1 snRNP binding [63,64]. Structural insights into cross-exon to cross-intron spliceosome switches are consistent with this idea, revealing that the pre-B complex is a convergence point for 5′ splice site selection in both the cross-exon and cross-intron pathways [27]. Together, these findings suggest that *in vivo* U1 snRNA pairing cannot solely determine 5′ splice site selection. Instead, human 5′ splice sites are probably selected at the pre-B stage through a coordinated transfer process from U1 to U5 and U6 snRNAs.

## 7. 5′ splice site interactions influence 3′ splice site usage

The impact of the two 5′ splice site classes is not restricted to 5′ splice site selection or the splicing efficiency of an intron. The helix formed between the 5′ splice site and the U6 snRNA ACAGA box is close to the active site that cleaves the 5′ splice site but also, with some structural rearrangement, forms a receptor onto which the 3′ splice site docks for exon ligation [65] as shown in figure 1. Consistent with this, the loss of U6 snRNA m^6^A modification in *Arabidopsis* is associated with thousands of changes in 3′ splice site usage that can be explained by the interaction of the U6 snRNA ACAGA box with 5′ splice sites [36]. Among the events affected is the selection of so-called NAGNAG (*n* = A, C, G or U) acceptors, where alternative 3′ splice sites are positioned almost directly adjacent to one another and in frame [66]. Tissue-specific regulation of NAGNAG acceptors has been identified in different species, but it is unclear how this regulation can occur [66]. Among possible regulators of differential NAGNAG usage are factors associated with the spliceosome during exon ligation [67,68]. In addition, modulation of interaction potentials between 5′ splice site sequences and the U6 snRNA ACAGA box can influence NAGNAG usage [36]. This is important because NAGNAGs impact changes in exon boundaries and protein evolution [66], underlining the broader potential influence of specific 5′ splice site classes on the evolution and regulation of alternative splicing.

## 8. How widespread are the two major classes of 5′ splice sites?

Evidence for two major classes of 5′ splice sites was first reported in developmentally complex species such as *Arabidopsis*, *C. elegans*, *Drosophila*, zebrafish and humans [36]. Since patterns of splicing complexity differ between species, it raises the question of how widespread two major classes of 5′ splice sites might be. Splicing in humans is characterized by a relatively high density of introns per gene, extensive patterns of alternative splicing, variable splicing signal sequences and two major classes of 5′ splice sites. In contrast, splicing has been simplified in many fungal species, including *S. cerevisiae*, which have evolved simplified development patterns and spend most or all of their life cycle in a unicellular form as yeasts [69]. In these species, many introns have been lost, such that most genes lack introns, alternative splice site selection is rare and many genes encoding splicing factors have also been lost [2,70–74].

*S. cerevisiae* is a member of the Saccharomycotina clade of fungi, which includes other species that appear to have simplified splicing [74]. Analysis of the introns encoded in more than 200 Saccharomycotina species’ genomes revealed that the most variable splicing signal was base preference at the 5′ splice site +4 position [49]. In species with reduced intron number (less than approx. 600), a 5′ splice site sequence preference switch from +4A to +4U has evolved on multiple independent occasions. Inter-species association mapping demonstrates that these switches in splice site sequence preference are associated with the loss of genes encoding METTL16 and SNRNP27K orthologues [49]. Consequently, these splice site changes over evolutionary time are consistent with splice sites sensitized to METTL16 and SNRNP27K mutations in diverse species and biophysical data demonstrating that m^6^A : U pairs tend to be unfavourable [36,38,39,43,49,53,54]. There is no clear evidence of two major classes of 5′ splice sites in the species with reduced intron number. Instead, the interaction potentials of U5 snRNA stem loop 1, and the U6 snRNA ACAGA box are mostly positively correlated, consistent with these species' more invariant splicing signals [2,49,75]. In contrast, in species with 5′ splice site +4A preference, there is a propensity for the interaction potentials of U5 snRNA loop 1 and the U6 snRNA ACAGA box to become increasingly anti-correlated (which characterizes the two 5′ splice site classes) as intron number increases [49]. Therefore, the simplification of splicing in *S. cerevisiae* and other fungi, characterized by the loss of many introns, is associated with the loss of two major classes of 5′ splice sites and the gain of a more unified consensus sequence [49].

## 9. The two classes of 5′ splice sites have regulatory potential

Comparison of annotated pairs of alternative 5′ splice sites in *Arabidopsis* and in humans reveals that if one had a strong interaction potential with the U6 snRNA ACAGA box then the alternative site was more likely to have a strong interaction potential with U5 snRNA loop 1 [36]. Consequently, the two 5′ splice site classes could establish a feature difference that contributes to alternative splicing choices.

Little is known about the regulation of U6 snRNA m^6^A modification or core splicing factors that act during the transfer of the 5′ splice site to U5 and U6 snRNA for selection. It is currently unknown if the multiple copies of U6 snRNA genes found in developmentally complex eukaryotes have different or regulated m^6^A levels [76]. However, two *Arabidopsis* genome-wide association studies have linked flowering time variation to the METTL16 orthologue, FIO1 [77,78], indicating possible variation in U6 snRNA m^6^A modification stoichiometry. Identifying proteins functionally involved in the transfer of the 5′ splice site to U6 snRNA should reveal further potential targets of regulation and circumstances in which they are subject to condition-specific control.

## 10. Why are there two sub-classes of the 5′ splice site?

Evolutionary analyses indicate that intron-dense genes with degenerate splice site signals are ancient and a likely feature of the last eukaryote common ancestor [2]. The consensus sequences for the two 5′ splice site classes are inherently weak as judged by the splicing efficiency of all possible 5′ splice site sequences measured in different human cell types [79]. This feature probably enables plasticity in splice site use since plasticity inherently means weaker splicing signals. Such plasticity facilitates tolerance to some splice site mutations that may lead to relatively small changes in mis-splicing or cryptic splicing, which persist through drift despite proofreading mechanisms [80]. However, in specific conditions, such small changes can, in turn, provide substrates that lead to the evolutionary selection of new beneficial traits [4]. The cooperative and compensatory activity of U5 and U6 snRNA interactions at 5′ splice sites probably facilitate this plasticity by buffering or compensating mutations at either side of the splice site. Many changes in 5′ splice site usage found in *Arabidopsis* METTL16 orthologue mutants involve very local switches from sites with a strong interaction potential with the U6 snRNA ACAGA box //GURAGGU, to sites with a strong interaction potential with U5 snRNA loop 1 in the same stretch of sequence: GURAG//GU, indicating relatively simple routes for the evolutionary change of one 5′ splice site class to another [81]. Significantly, mutations to the different splice site classes could drive alternative splicing choices without requiring mutation of either core spliceosomal components or the evolution of regulatory factors that modulate initial recruitment of the spliceosome to the general neighbourhood of splice sites. This mechanism could allow the evolution of new functional exon boundaries in specific genes under selective pressure without causing general splicing disruption in the wider transcriptome. Such evolution is indeed observed for NAGNAG 3′ splice site boundaries, which are also indirectly influenced by alternative 5′ splice site selection mediated by METTL16 activity [36].

Splice site sequences could be degenerate but not necessarily separate into two sub-classes defined by a preferred interaction potential with either U5 snRNA loop 1 or the U6 snRNA ACAGA box. The molecular mechanisms for 5′ splice site transfer revealed by cryo-EM structures indicate that sub-optimal interactions with either U5 or U6 snRNA create a potential equilibrium between the pre-B and B complexes [27] (figure 4). Therefore, the link between plasticity, regulation and the persistence of two 5′ splice site classes through time may be that alternative 5′ splice site usage is facilitated by suboptimal interactions between U1 snRNA and the 5′ splice site in the precursor pre-B state and between the 5′ splice site and either U5 or U6 snRNA in the product B state. Consequently, by modulating the strength of these interactions (figure 5), trans-acting factors may regulate the equilibrium between the stability of the pre-B and B complexes to achieve different splicing outcomes.

## 11. Two splice site classes underline the role of U5 and U6 snRNA in splice site selection

Early *in vitro* splicing analysis of test transcripts led to the idea that the commitment to 5′ and 3′ splice site selection occurred in the spliceosomal E complex [82] or A complex [83,84] before the recruitment of U5 and U6 snRNA to the pre-mRNA. However, the combination of global RNA sequencing, which reveals splicing in diverse sequence contexts, and cryo-EM analyses, which reveals many more spliceosome conformations than native gel electrophoresis can resolve, has led to the revision of this idea. Crucially, 3′ splice site selection involves pairing of all splice sites late during the catalytic stage in C* [67], suggesting that final commitment to the 3′ splice site used can only occur during exon ligation. Therefore, splice site selection can still be regulated late in the splicing cycle, during catalysis [25]. Indeed, several proteins, which are not recruited to human C* spliceosomes until after the first cleavage reaction, influence alternative 3′ splice site selection *in vivo* [68]. Since U5 and U6 snRNA affect 5′ splice site selection, commitment to a 5′ splice site cannot exclusively occur before their recruitment.

Massively parallel splicing assays in human cells of all possible 32 768 unique 5′ splice site sequences (NNN/GYNNNN) show that splicing efficiency is mainly governed by the 5′ splice site sequence but with context-specific effects [79]. However, the free energy of U1/5′ splice site base pairing was the least predictive method of splicing efficiency [79]. Different U1 snRNA base pairing registers, base modifications or the influence of protein interactions may explain, in part, these distinctions [24], but so too might the interactions of 5′ splice sites with U5 and U6 snRNA. Strikingly, *in vitro* selection of functional human 5′ splice sites identifies a sequence closely matching the established consensus, but this can be detected even when selection is performed in extract with a deleted U1 snRNA 5′ end [85]. In humans, U1 snRNA is by far the most abundant spliceosomal UsnRNA [86], and its flexibility in the sequence and registers of RNAs it pairs with indicate that U1 snRNA cannot be the rate-limiting, determining step in splice site selection. Indeed, in *S. cerevisiae,* proofreading of branching by the ATPase Prp28 depends primarily on the competition with the stability of 5′ splice site interactions with U6 (figure 1), rather than U1 snRNA, consistent with a key role for U6, rather than U1 snRNA, in selecting the cleaved 5′ splice site [51].

Spliceosomes and splicesosomal introns probably derive from the evolutionary fragmentation of group II introns into splicing functions mediated by *trans*-acting UsnRNAs and proteins [2,29,87,88]. While the functional equivalents of the U6 snRNA ACAGA box, U5 snRNA loop 1 and U6-U2 snRNA interactions that form the active site, can be traced in group II introns, U1 and U4 snRNA cannot [29]. Therefore, U1 snRNA probably evolved later to help identify 5′ splice sites that may already have separated into two sub-classes. Splicing in the unicellular red alga *Cyanidioschyzon merolae* occurs without genes encoding U1 snRNA or conserved U1 snRNP components [89], indicating that U1 snRNA can be gained and U1 snRNA can be lost.

The architecture of two 5′ splice site classes in eukaryote genome sequences and their sensitization by factors that function during the transfer of 5′ splice sites to U6 snRNA mirrors structural insight, revealing that commitment to 5′ splice site usage primarily occurs during the handover from U1 to U6 snRNA [27]. Since it has already been established that U5 snRNA loop 1 and the U6 snRNA ACAGA box affect 5′ splice site selection [18–22], the recent studies on two splice site classes indicate that U5 and U6 snRNA functions are separable major influences on the diversity of 5′ splice site sequences. Indeed, the partially redundant interactions of U5 and U6 snRNA with 5′ splice sites may account for two relatively weak splice site classes. Therefore, two 5′ splice site classes underline a crucial role for U5 and U6 snRNA in splice site selection, which is not necessarily the memory or maintenance of sites bound by U1 snRNA.

## 12. So, what is the 5′ splice site consensus?

In species with complex splicing phenotypes, such as humans, the 5′ splice consensus AG//GURAG masks the presence of two major constituent sub-classes, AG//GUNNN and NN//GURAG. In other words, the established 5′ splice site consensus can be split into two. In species where splicing has been simplified, such as *S. cerevisiae*, these two 5′ splice site classes are lost, and more invariant splicing signal phenotypes have evolved instead. Still, not all splicing happens at introns with GU-AG borders, so are two 5′ splice site classes detected in other contexts?

GC 5′ splice sites are the most frequently occurring exception to the GU 5′ splice site boundary rule but constitute only 0.87% of human 5′ splice sites [35]. The rarity of +2C is probably because the canonical +2U forms part of a network of non-Watson–Crick RNA–RNA interactions between the 5′ splice site GU, 3′ splice site AG and branchpoint adenosine that proofreads the splicing reaction [65]. GC sites in human 5′ splice sites have a consensus G at −1 and +5, indicating they do not fall into the two sub-classes we define here, but this is consistent with the idea that cooperative interactions between U5 snRNA loop 1 and the U6 snRNA ACAGA box compensate for the negative impact of +2C on splicing efficiency. Consistent with this idea, studies with *S. cerevisiae* suggest that changes at +3 or +5 may require additional stabilization by Hub1/UBL5, which would stabilize interactions with U5 snRNA [90]. *BRCA2* mutations perhaps exemplify the importance of this cooperative activity: Intron 17 of the *BRCA2* gene has a GC 5′ splice site and a −1G and +5G matching the consensus of human GC 5′ splice sites [35]. Mutations of the −1G to either A or C and of +5G to A have been found in breast cancer samples [91–93] and disrupt the splicing of *BRCA2* mRNA [79]. The splicing of these mutated 5′ splice sites cannot be rescued by the expression of modified U1 snRNA molecules with increased complementarity to 5′ splice site positions +7 and +8 [79], raising the question of whether compensatory mutations in U5 or U6 snRNAs might instead rescue −1G and +5G mutations, respectively.

Approximately 0.37% of human introns are processed by minor spliceosomes [35]. The only UsnRNA shared between major and minor spliceosomes is U5, which, along with U11, U12, U4atac and U6atac snRNAs, forms dynamic RNA networks within minor spliceosomes to process U12-dependent introns. Two sub-classes of 5′ splice sites are not apparent in U12-dependent introns. Instead, a more invariant U6atac/5′ splice site base-pairing appears to constitute a strong intron splicing signal that facilitates selection stabilized by minor spliceosome proteins [94]. Base pairing to U11 snRNA does not absolutely define the 5′ splice site for minor introns [95].

## 13. Conclusion and outlook

Two major classes of 5′ splice sites are a fundamental but overlooked feature of eukaryotic genome architecture subject to evolutionary change. After nearly 50 years of studying pre-mRNA splicing, we are only beginning to address the impact of separating the 5′ splice site consensus based on specific molecular interactions with spliceosome components.

Cryo-EM structures of spliceosomes have transformed our understanding of splicing mechanisms [23]. In the future, higher resolution cryo-EM analysis, with different substrates and genetic backgrounds, should reveal precisely how m^6^A modification of the U6 snRNA ACAGA box facilitates splice site selection and explain why variation in base preference at the 5′ splice site +4 position is so important [49]. Examining distinctions in spliceosome composition during the processing of a broader range of pre-mRNAs that reflect different 5′ splice site classes should also distinguish whether U5 or U6 snRNA interactions lead when selecting different splice site classes. Likewise, global splicing analysis using RNA sequencing has provided network views of alternative splicing in different biological contexts [96]. It will be interesting to examine the extent to which alternative splicing involves two major 5′ splice site classes using bioinformatic approaches that distinguish such sites [36,47].

Mutations that disrupt splicing are an important fraction of inherited genetic variation that cause human disease [97,98]. Rebalancing the consideration of the impact that 5′ splice site mutations have on not only U1 snRNA interactions but also the roles of U5 and U6 snRNAs (and other spliceosome elements that modulate RNA–RNA interactions during 5′ splice site transfer) may inform the design of new therapeutic approaches.

## Data Availability

This article has no additional data.
